# APOEε4 potentiates the relationship between amyloid-β and tau pathologies

**DOI:** 10.1038/s41380-020-0688-6

**Published:** 2020-03-11

**Authors:** Joseph Therriault, Andrea L. Benedet, Tharick A. Pascoal, Sulantha Mathotaarachchi, Melissa Savard, Mira Chamoun, Emilie Thomas, Min Su Kang, Firoza Lussier, Cecile Tissot, Jean-Paul Soucy, Gassan Massarweh, Soham Rej, Paramita Saha-Chaudhuri, Judes Poirier, Serge Gauthier, Pedro Rosa-Neto, for the Alzheimer’s Disease Neuroimaging Initiative

**Affiliations:** 1 Translational Neuroimaging Laboratory, The McGill University Research Centre for Studies in Aging, Douglas Hospital, McGill University, Montreal, QC Canada; 2 Department of Neurology and Neurosurgery, McGill University, Montreal, QC Canada; 3 Montreal Neurological Institute, Montreal, QC Canada; 4 Department of Radiochemistry, McGill University, Montreal, QC Canada; 5 Department of Psychiatry, McGill University, Montreal, QC Canada; 6 Department of Epidemiology and Biostatistics, McGill University, Montreal, QC Canada

**Keywords:** Neuroscience, Diseases

## Abstract

*APOEε4* is the most well-established genetic risk factor for sporadic Alzheimer’s disease and is associated with cerebral amyloid-β. However, the association between *APOEε4* and tau pathology, the other major proteinopathy of Alzheimer’s disease, has been controversial. Here, we sought to determine whether the relationship between *APOEε4* and tau pathology is determined by local interactions with amyloid-β. We examined three independent samples of cognitively unimpaired, mild cognitive impairment and Alzheimer’s disease subjects: (1) 211 participants who underwent tau-PET with [^18^F]MK6240 and amyloid-PET with [^18^F]AZD4694, (2) 264 individuals who underwent tau-PET with [^18^F]Flortaucipir and amyloid-PET with [^18^F]Florbetapir and (3) 487 individuals who underwent lumbar puncture and amyloid-PET with [^18^F]Florbetapir. Using a novel analytical framework, we applied voxel-wise regression models to assess the interactive effect of *APOEε4* and amyloid-β on tau load, independently of age and clinical diagnosis. We found that the interaction effect between *APOEε4* and amyloid-β, rather than the sum of their independent effects, was related to increased tau load in Alzheimer’s disease-vulnerable regions. The interaction between one *APOEε4* allele and amyloid-β was related to increased tau load, while the interaction between amyloid-β and two *APOEε4* alleles was related to a more widespread pattern of tau aggregation. Our results contribute to an emerging framework in which the elevated risk of developing dementia conferred by *APOEε4* genotype involves mechanisms associated with both amyloid-β and tau aggregation. These results may have implications for future disease-modifying therapeutic trials targeting amyloid or tau pathologies.

## Introduction

The mechanisms by which *APOEε4* imposes a genetic risk factor for sporadic Alzheimer’s disease are not fully understood. While early work linked *APOEε4* with both amyloid-β and tau pathologies, much of the focus of the role of *APOEε4* has been in relation to amyloid-β [1]. The *APOEε4* allele is associated with increased production [2, 3] as well as diminished clearance of cerebral amyloid-β [4, 5]. Individuals with the *APOEε4* genotype also demonstrate increased amyloid-β PET uptake [6], with amyloid positivity beginning earlier in life in *APOEε4* carriers than noncarriers [7].

Together, these findings are interpreted to suggest that the mechanism through which the *APOEε4* allele confers risk for Alzheimer’s disease is by leading to increased cerebral amyloid-β burden, considered to be the central pathological event in Alzheimer’s disease [8]. However, recent work has demonstrated that the *APOEε4* allele is also related to inflammation and neurodegeneration in mouse models, as well as faster disease progression in humans [9]. Furthermore, previous observational studies have reported that the *APOEε4* allele modifies the relationship between amyloid-β and cognitive decline [10–13], though the precise mechanisms underlying this relationship remain unclear.

Given its close association with cognitive deficits [14–16], aggregation of tau pathology presents a potential mechanism for *APOEε4* modifying the relationship between amyloid-β and cognitive decline [17]. While recent tau-PET studies have reported inconsistent effects of *APOEε4* on tau-PET uptake [14, 15, 18], no studies have assessed whether *APOEε4* potentiates the relationship between amyloid-β and tau pathologies. Thus, we aimed to determine if tau pathology depends on the synergistic interaction between *APOEε4* and amyloid-β, rather than the sum of their independent effects. We hypothesize that *APOEε4* synergistically interacts with amyloid-β to drive tau aggregation.

## Materials and methods

### Participants

#### TRIAD

The Translational Biomarkers in Aging and Dementia (TRIAD) cohort aims at modeling biomarker trajectories and interactions as drivers of dementia. TRIAD was launched in 2017 as part of the McGill Centre for Studies in Aging. We assessed cognitively unimpaired (*n* = 138), mild cognitive impairment (*n* = 26), and Alzheimer’s disease dementia (*n* = 47) subjects who underwent amyloid-β PET with [^18^F]AZD4694, tau-PET with [^18^F]MK6240, structural MRI and genotyping for *APOEε4*. All subjects had detailed clinical assessments including Mini-Mental State Examination (MMSE), Clinical Dementia Rating (CDR), and cerebrovascular disease risk with the Hachinski Ischemic scale [19]. Cognitively unimpaired controls had a CDR of 0, mild cognitive impairment subjects had a CDR of 0.5, and Alzheimer’s disease participants had a CDR of 1 or 2, in addition to meeting standard diagnostic criteria [20]. Similar to other large-scale cohort studies of aging and Alzheimer’s disease [13], the TRIAD Cohort is enriched for *APOEε4* carriers. This study’s protocol was approved by the Institutional Review Board at McGill University and informed consent was obtained from every subject or their caregiver.

#### ADNI

Data used in the preparation of this article were obtained from the Alzheimer’s Disease Neuroimaging Initiative (ADNI) database (adni.loni.usc.edu). The ADNI was launched in 2003 as a public–private partnership led by principal investigator Michael W. Weiner, MD. The primary goal of ADNI has been to test whether serial MRI, PET, other biological markers, and clinical and neuropsychological assessment can be combined to measure the progression of mild cognitive impairment and early Alzheimer’s disease. In this study, we assessed cognitively normal (*n* = 157), amnestic mild cognitive impairment (*n* = 83), and Alzheimer’s disease dementia (*n* = 24) individuals from ADNI cohort who underwent amyloid-β PET with [^18^F]Florbetapir, tau-PET with [^18^F]Flortaucipir (also known as [^18^F]T807 and [^18^F]AV1451), structural MRI and genotyping for *APOEε4*. We also examined a third independent sample of cognitively normal (*n* = 104), amnestic mild cognitive impairment (*n* = 283), and Alzheimer’s disease (*n* = 100) individuals from ADNI cohort who underwent amyloid-β PET with [^18^F]Florbetapir, lumbar puncture, structural MRI, and genotyping for *APOEε4*. Cognitively normal controls had a CDR of 0, MCI subjects had a CDR of 0.5, and Alzheimer’s disease participants had a CDR of 1 or greater in addition to meeting standard diagnostic criteria [20]. Full information regarding the ADNI inclusion and exclusion criteria can be accessed at http://adni.loni.usc.edu/ (accessed April 2019). The ADNI study was approved by the Institutional Review boards of all of the participating institutions. Informed written consent was obtained from all participants at each site.

### Genetic and CSF analyses

#### TRIAD

Determination of *APOE* genotypes for subjects enrolled in the TRIAD cohort was performed using the polymerase chain reaction amplification technique, followed by restriction enzyme digestion, standard gel resolution, and visualization processes. Full details of this procedure can be found elsewhere [21].

#### ADNI

Determination of *APOE* genotypes for ADNI subjects took place at the University of Pennsylvania Alzheimer’s Disease Biomarker Laboratory. CSF measurements of tau phosphorylated at threonine 181 were assessed using the multiplex xMAP Luminex platform (Luminex, Austin, TX, USA) with INNOBIA AlzBio3 (Innogenetics, Ghent, Belgium) immunoassay kit-based reagents. The CSF biomarker data sets used in this study were obtained from the ADNI files ‘UPENNBIOMK5-8.csv’. We considered a subject positive for tau hyperphosphorylation if the CSF p-tau value was above the ADNI published threshold (0.23 pg/mL) [22, 23]. Complete details of CSF methods employed in ADNI can be accessed at http://adni.loni.usc.edu/data-samples/clinical-data/.

### PET image acquisition and processing

#### TRIAD

All subjects had a T1-weighted MRI which was used for coregistration. PET scans were acquired with a Siemens High Resolution Research Tomograph. [^18^F]MK6240 images were acquired 90–110 min post injection and scans were reconstructed with the OSEM algorithm on a 4D volume with 4 frames (4 × 300 s) [24]. [^18^F]AZD4694 images were acquired 40–70 min post injection and scans were reconstructed with the OSEM algorithm on a 4D volume with three frames (3 × 600 s) [25]. Immediately following each PET acquisition, a 6-min transmission scan was conducted with a rotating ^137^Cs point source for attenuation correction. Furthermore, the images underwent correction for dead time, decay, and random and scattered coincidences. T1-weighted images were nonuniformity and field-distortion corrected and processed using an in-house pipeline. Then, PET images were automatically registered to the T1-weighted image space, and the T1-weighted images were linearly and nonlinearly registered to the ADNI template space. Next, a PET nonlinear registration was performed using the linear and nonlinear transformations from the T1-weighted image to the ADNI template space and the PET to T1-weighted image registration. The PET images were spatially smoothed to achieve a final resolution of 8 mm full-width at half maximum. PET image partial volume correction was carried out using the PETPVC toolbox [26]. The region-based voxel-wise correction technique was used to perform partial volume correction using ten tissue priors with a gaussian kernel with the FWHM of 2.4 mm [27]. [^18^F]MK6240 standardized uptake value ratio (SUVR) maps were generated using the inferior cerebellar gray matter as a reference region and [^18^F]AZD4694 SUVR maps were generated using the cerebellar gray matter as a reference region. A global [^18^F]AZD4694 SUVR value was estimated for each participant by averaging the SUVR from the precuneus, prefrontal, orbitofrontal, parietal, temporal, anterior, and posterior cingulate cortices.

#### ADNI

Full information regarding acquisition of PET data in ADNI is provided at http://adni.loni.usc.edu/data-samples/pet/. Preprocessed PET images downloaded from ADNI underwent spatial normalization to the ADNI standardized space using the transformations of PET native to MRI native space and MRI native to the ADNI space. Partial volume correction was carried out using the PETPVC toolbox [26] described above in an effort to diminish off-target binding to the choroid plexus. [^18^F]Flortaucipir SUVR maps were generated using the inferior cerebellar gray matter as a reference region [28] and [^18^F]Florbetapir SUVR maps were generated using the cerebellar gray matter as a reference region. A global [^18^F]Florbetapir SUVR value was estimated for each participant by averaging the SUVR from the precuneus, prefrontal, orbitofrontal, parietal, temporal, anterior, and posterior cingulate cortices.

### Statistical analyses

To measure tau pathology in vivo, we used CSF and PET measurements. Previous studies have demonstrated associations between CSF p-tau and tau-PET uptake in Alzheimer’s disease-related brain regions [29–31]. The primary outcome measure of the study was tau pathology as measured by voxel-wise [^18^F]MK6240 SUVR (TRIAD cohort), [^18^F]Flortaucipir SUVR (ADNI tau-PET cohort), and CSF phosphorylated tau (ADNI CSF cohort). Three independent samples were investigated cross-sectionally: (1) the McGill cohort assessed with [^18^F]MK6240 and [^18^F]AZD4694 (2) an ADNI cohort assessed with [^18^F]Flortaucipir and [^18^F]Florbetapir and (3) an ADNI cohort assessed with [^18^F]Florbetapir and lumber puncture for CSF phosphorylated tau. In each cohort, we tested the hypothesis that the synergistic interaction between amyloid-β and *APOEε4* is related to tau pathology, i.e., the interaction between amyloid-β and *APOEε4* is greater than the sum of the additive effects of amyloid-β and *APOEε4* [32, 33]. In each cohort, we measured the effect of one *APOEε4* allele or two *APOEε4* alleles, meaning that the comparison is to individuals who do not carry *an APOEε4* allele. The results displayed in this manuscript are multiple comparisons corrected *t*-values (Random Field Theory with a cluster threshold of *p* < 0.005). From RFT-corrected significant clusters, we subsequently extracted the beta estimates and standard errors.

Baseline demographic and clinical data were assessed using *t* tests and *χ*^2^ tests. Neuroimaging analyses were carried out using the VoxelStats toolbox (https://github.com/sulantha2006/VoxelStats), a MATLAB-based analytical framework that allows for the execution of multimodal voxel-wise neuroimaging analyses [34]. Other statistical analyses were performed using the R Statistical Software Package version 3.0.2 (http://www.r-project.org/). In models with interaction terms, predictor variables were centered on the mean for improved interpretability of coefficients and to improve numerical stability for estimation associated with multicollinearity [35]. Given the large number of covariates in the statistical models, model diagnostics were carried out using the car package in R to determine the presence of multicollinearity. We computed the Variance Inflation Factor (VIF), a measurement of how much variance in regression coefficients are inflated due to multicollinearity in the statistical models [36]. All neuroimaging analyses described below were repeated using partial volume corrected data. To provide further confirmation that the interaction term is statistically significant, we computed the absolute values of the beta estimates from RFT-corrected statistically significant clusters, e.g., absolute value of (beta(X1) + beta(X2) + beta(X1 × X2)) > absolute value of (beta(X1) + beta(X2)) [32, 33].

In the TRIAD cohort, the voxel-based interaction model outlined below was built to test whether main and interactive effects between *APOEε4* carriership and [^18^F]AZD4694 SUVR at every voxel are associated with [^18^F]MK6240 uptake. To ensure that the results are not driven by an effect of clinical status, we adjusted the model for clinical diagnosis. The model was also adjusted for age. Because *APOEε4* is related to amyloid-PET uptake, amyloid-β was included as a covariate in every analysis. Statistical parametric maps were corrected for multiple comparisons using a random field theory [37] threshold with a cluster threshold of *P* < 0.005. The analysis was repeated using partial volume corrected data. The analysis was also repeated excluding a subject with the *ε*2/*ε*4 genotype.

$${\mathrm{Tau}}\;{\mathrm{PET}}\;{\mathrm{SUVR}} = \,	\beta _0 + \beta _1\,\left( {{\mathrm{Amyloid}}\;{\mathrm{PET}}\;{\mathrm{SUVR}}} \right)\\ 	 \!+ \,\beta _2\,\left( {APOE_{\varepsilon 4}} \right) +\, \beta _3\,\left( {{\mathrm{Amyloid}}\;{\mathrm{PET}}\;{\mathrm{SUVR}}\;x\;APOE_{\varepsilon 4}} \right) \\ 	\! +\, \beta _4\,\left( {\mathrm{Clinical}}\;{\mathrm{Status}} \right) + \beta _5\,({\mathrm{Age}})\; + \; \in.$$

Next, we aimed to investigate a possible gene-dose relationship in the ADNI database, a larger cohort containing more homozygous *APOEε4* carriers. In these gene-dose analyses, *APOE* status was treated as a categorical variable with three levels (Noncarriers < Heterozygotes < Homozygotes). No individuals in the ADNI tau-PET cohort were also included in the ADNI CSF analyses. This model was also adjusted for cerebral amyloid-β, age, and clinical status. Statistical parametric maps were corrected for multiple comparisons using a random field theory [37] threshold with a cluster threshold of *P* < 0.005. The analysis was repeated using partial volume corrected data.

$${\mathrm{Tau}}\;{\mathrm{PET}}\;{\mathrm{SUVR}} = 	\;\beta _0 + \beta _1\,\left( {{\mathrm{Amyloid}}\;{\mathrm{PET}}\;{\mathrm{SUVR}}} \right) \\ 	 \!+ \beta _2\,\left( {APOE_{\varepsilon 3\varepsilon 4}} \right)\\ + \beta _3\,\left( {APOE_{\varepsilon 4\varepsilon 4}} \right) \\ 	 \!+ \beta _4\,\left( {{\mathrm{Amyloid}}\;{\mathrm{PET}}\;{\mathrm{SUVR}}\;x\;APOE_{\varepsilon 3\varepsilon 4}} \right)\\ 	 \!+ \beta _5\,\left( {{\mathrm{Amyloid}}\;{\mathrm{PET}}\;{\mathrm{SUVR}}\;x\;APOE_{\varepsilon 4\varepsilon 4}} \right) \\ 	 \!+ \beta _6\,\left( {Clinical\;Status} \right) + \beta _7\,(Age) \,+ \in.$$

In order to gain a better understanding of the similarities between cohorts, we repeated the analyses in ADNI using the same *APOEε4* carrier/noncarrier framework as conducted in the TRIAD cohort.

We followed up the tau-PET analyses by testing the hypothesis in an independent sample of 487 individuals who underwent amyloid-β PET with [^18^F]Florbetapir and lumbar puncture, with CSF phosphorylated tau as an outcome measure. No individuals in the ADNI CSF cohort were also included in the ADNI tau-PET analyses. *APOE* status was treated in a dose-dependent manner and the model was adjusted for age, clinical diagnosis, and the main effect of amyloid-β.

$${\mathrm{CSF}}\;{\mathrm{phosphorylated}}\;{\mathrm{Tau}} =	\,\, \beta _0 + \beta _1\,\left( {{\mathrm{Amyloid}}\;{\mathrm{PET}}\;{\mathrm{SUVR}}} \right) \\ 	+ \beta _2\, \left( {APOE_{\varepsilon 3\varepsilon 4}} \right)\\ + \beta _3\,\left( {APOE_{\varepsilon 4\varepsilon 4}} \right) \\ 	 + \beta _4\,\left( {{\mathrm{Amyloid}}\;{\mathrm{PET}}\;{\mathrm{SUVR}}\;x\;APOE_{\varepsilon 3\varepsilon 4}} \right)\\ \\ 	 + \beta _5\,\left( {{\mathrm{Amyloid}}\;{\mathrm{PET}}\;{\mathrm{SUVR}}\;x\;APOE_{\varepsilon 4\varepsilon 4}} \right) \\ 	 + \beta _6\,\left( {\mathrm{Clinical}}\;{\mathrm{Status}} \right) + \beta _7\,(Age) \,+ \in.$$

## Results

Demographic and clinical information for the three samples examined in this study is summarized in Table 1. VIFs for all variables in all cohorts are presented in Supplementary table 1. VIFs for all variables were below 4, indicating that problematic levels of multicollinearity are not present in our analyses [36]. Table 2 presents the estimates of main and interactive effects of amyloid-PET and *APOEε4* on tau pathology in the three independent samples. Standardized estimates are presented in Supplementary Table 2. The brain regions displayed in Table 2 correspond to regions that were statistically significant after correction for multiple comparisons.

**Table 1:** Demographic and key characteristics of the samples.

(A) TRIAD tau-PET cohort	CN	MCI	*P* value	AD	*P* value
No.	138	26	**–**	47	**–**
Age, years, mean (SD)	68.32 (11.54)	74.4 (5.45)	0.007	66.63 (11.34)	0.28
Male, no. (%)	53 (38)	13 (50)	0.3	20 (43)	0.61
Education, years, mean (SD)	15.17 (3.77)	14.36 (3.79)	0.84	14.89 (3.72)	0.92
*APOE ε4 heterozygous*, %	43 (31)	9 (34)	0.21	20 (43)	0.08
*APOE ε4 homozygous*, %	1 (0.7)	1 (4)	0.17	5 (10)	0.002
MMSE, mean (SD)	29.05 (1.25)	27.13 (2.39)	<0.0001	19.1 (7.31)	<0.0001
CDR SoB, mean (SD)	0.18 (0.45)	1.47 (1.23)	<0.0001	6.48 (4.08)	<0.0001
[^18^F]AZD4694 SUVR, (SD)	1.48 (0.42)	1.86 (0.54)	0.0001	2.42 (0.63)	<0.0001
Braak 1&2 [^18^F]MK6240 SUVR, (SD)	0.98 (0.24)	1.32 (0.55)	<0.0001	1.82 (0.63)	<0.0001
Braak 3&4 [^18^F]MK6240 SUVR, (SD)	1.09 (0.23)	1.41 (0.62)	<0.0001	2.73 (1.21)	<0.0001
Braak 5&6 [^18^F]MK6240 SUVR, (SD)	1.12 (0.21)	1.31 (0.38)	<0.0001	2.55 (1.23)	<0.0001

(B) ADNI tau-PET cohort	CN	MCI	*P* value	AD	*P* value
No.	157	83	**–**	24	**–**
Age, years, mean (SD)	70.98 (5.91)	70.57 (7.09)	0.63	74.11 (7.65)	0.02
Male, no. (%)	71 (45)	49 (59)	0.04	12 (50)	0.66
Education, years, mean (SD)	16.65 (2.5)	15.84 (2.85)	0.02	16.26 (2.51)	0.47
*APOE ε4 heterozygous*, %	44 (28)	13 (15.6)	0.08	9 (37.5)	0.19
*APOE ε4 homozygous*, %	5 (3.1)	11 (13.3)	0.008	3 (12.5)	0.019
MMSE, mean (SD)	28.97 (1.33)	28.05 (2.15)	<0.0001	19.67 (5.28)	<0.0001
CDR SoB, mean (SD)	0.009 (0.51)	1.46 (0.93)	<0.0001	7.18 (2.67)	<0.0001
[^18^F]Florbetapir SUVR, (SD)	1.2 (0.22)	1.26 (0.29)	0.07	1.47 (0.22)	<0.0001
Braak 1&2 [^18^F]Flortaucipir SUVR, (SD)	1.14 (0.13)	1.21 (0.2)	<0.0001	1.4 (0.233)	<0.0001
Braak 3&4 [^18^F] Flortaucipir SUVR, (SD)	1.08 (0.09)	1.15 (0.2)	<0.0001	1.46 (0.43)	<0.0001
Braak 5&6 [^18^F] Flortaucipir SUVR, (SD)	0.99 (0.09)	1.06 (0.18)	<0.0001	1.25 (0.34)	<0.0001

(C) ADNI lumbar puncture cohort	CN	MCI	*P* value	AD	*P* value
No.	104	283	**–**	100	**–**
Age, years, mean (SD)	73.66 (6.41)	72.1 (7.31)	0.06	74.21 (8.06)	0.59
Male, no. (%)	54 (51.9)	153 (54.06)	0.14	61 (61)	0.19
Education, years, mean (SD)	16.6 (2.58)	16.15 (2.59)	0.13	15.85 (2.64)	0.04
*APOE ε4 heterozygous*, %	21 (20.19)	112 (39.58)	0.0001	48 (48)	0.0001
*APOE ε4 homozygous*, %	6 (5.76)	29 (10.25)	0.0001	18 (18)	0.0001
MMSE, mean (SD)	29.06 (1.34)	27.96 (2.09)	<0.0001	23.18 (5.5)	<0.0001
CDR SoB, mean (SD)	0.05 (0.16)	1.51 (0.9)	<0.0001	4.52 (1.74)	<0.0001
[^18^F]Florbetapir SUVR, (SD)	1.13 (0.24)	1.22 (0.18)	0.0002	1.36 (0.17)	<0.0001
CSF p-tau pg/mL, (SD)	20.47 (7.88)	26.63 (13.81)	0.002	37.89 (16.79)	<0.0001
CSF p-tau positive, *%*	30 (29)	146 (52)	<0.0001	82 (82)	<0.0001

CSF p-tau positivity is based on a published cutoff of 23 pg/mL.

*P* values indicate values assessed with independent samples *t* tests for each variable except sex and APOE *ε4* status, where contingency chi-square tests were performed. *P* values reported are for comparisons with cognitively normal subjects.

*MMSE* Mini-Mental State Examination, *CDR SoB* Clinical Dementia Rating Sum of Boxes; *SUVR* standardized uptake value ratio, *p-tau* phosphorylated tau, *CN* cognitively normal, *MCI* mild cognitive impairment, *AD* Alzheimer’s disease.

**Table 2:** Main and interactive effects of amyloid-PET and *APOEε4* on tau-PET uptake and CSF p-tau.

(A) TRIAD tau-PET cohort				
Brain region	Amyloid-PET main effect β estimate (SE)	APOE4 main effect β estimate (SE)	Total of amyloid-PET main effect β estimate and APOE4 main effect β estimate	Amyloid-PET × APOE4 interaction effect β estimate (SE)
Posterior cingulate	0.19 (0.1)	0.02 (0.09)	0.17	0.26 (0.15)
Precuneus	0.13 (0.11)	−0.03 (0.09)	0.10	0.23 (0.14)
Inferior parietal	0.29 (0.1)	−0.07 (0.09)	0.22	0.23 (0.14)
Medial prefrontal	0.20 (0.08)	−0.04 (0.07)	0.16	0.25 (0.13)
Occipital	0.25 (0.07)	−0.02 (0.07)	0.23	0.41 (0.1)

(B) ADNI tau-PET cohort				
Brain region	Amyloid-PET main effect β estimate (SE)	Single APOE4 Main Effect β Estimate (SE)	Total of amyloid-PET main effect β estimate and single APOE4 main effect β estimate	Amyloid-PET × Single APOE4 interaction effect β estimate (SE)
Posterior cingulate	0.16 (0.05)	−0.04 (0.03)	0.12	0.28 (0.1)
Lateral temporal	0.37 (0.06)	−0.03 (0.03)	0.33	0.36 (0.11)
Inferior parietal	0.21 (0.05)	−0.02 (0.04)	0.19	0.29 (0.11)
Orbitofrontal	0.18 (0.05)	−0.04 (0.03)	0.14	0.24 (0.08)
Temporooccipital	0.21 (0.06)	−0.01 (0.04)	0.20	0.44 (0.11)

(C) ADNI tau-PET cohort				
Brain region	Amyloid-PET main effect β estimate (SE)	Double APOE4 main effect β estimate (SE)	Total of amyloid-PET main effect β estimate and double APOE4 main effect β estimate	Amyloid-PET × Double APOE4 interaction effect β estimate (SE)
Posterior cingulate	0.21 (0.05)	−0.07 (0.06)	0.14	0.39 (0.13)
Lateral temporal	0.24 (0.05)	−0.001 (0.06)	0.24	0.49 (0.15)
Inferior parietal	0.18 (0.04)	0.006 (0.07)	0.17	0.48 (0.15)
Medial prefrontal	0.19 (0.05)	−0.07 (0.05)	0.12	0.38 (0.11)
Occipital	0.2 (0.05)	−0.01 (0.05)	0.19	0.39 (0.12)
Orbitofrontal	0.24 (0.04)	−0.06 (0.05)	0.18	0.48 (0.14)
Dorsolateral prefrontal	0.15 (0.04)	−0.01 (0.06)	0.14	0.43 (0.13)

(D) ADNI lumbar puncture cohort				
	Amyloid-PET main effect β estimate (SE)	Single APOE4 main effect β estimate (SE)	Total of amyloid-PET main effect β estimate and single APOE4 main effect β estimate	Amyloid-PET × Single APOE4 interaction effect β estimate (SE)
CSF p-tau	14.81 (3.53)	3.94 (1.28)	18.75	20.31 (6.59)

(E) ADNI lumbar puncture cohort				
	Amyloid-PET main effect β estimate (SE)	Double APOE4 main effect β estimate (SE)	Total of amyloid-PET main effect β estimate and double APOE4 main effect β estimate	Amyloid-PET × Double APOE4 interaction effect β estimate (SE)
CSF p-tau	14.81 (3.53)	3.87 (2.55)	18.68	33.01 (14.24)

It reports beta coefficients for main and interactive effects of amyloid-PET and *APOEε4* on tau. A–C: beta coefficients from brain regions where a significant synergistic effect of amyloid-PET and *APOEε4* on tau-PET was observed. D, E: Beta coefficients from global neocortical amyloid-PET and *APOEε4* on CSF p-tau. Standard errors are reported in parentheses. The amyloid-PET and *APOEε4* interaction effect estimate is greater than the sum of the individual main effects, indicating the presence of a synergistic interaction. Standard errors are reported in parentheses.

**Fig. 1:**
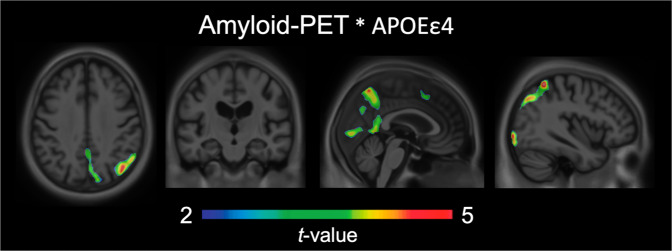
Cerebral tau aggregation depends on the synergistic interaction between amyloid-β and APOEε4. The synergistic interaction between [^18^F]AZD4694 and *APOEε4* carriership was related to increased [^18^F]MK6240 uptake in the posterior cingulate, precuneus, occipital, and inferior parietal cortices. T-statistical parametric maps were corrected for multiple comparisons using a Random Field Theory cluster threshold of *P* < 0.005, overlaid on the ADNI reference template. Age, clinical diagnosis, and amyloid-β SUVR were employed as covariates the model.

We tested the hypothesis that amyloid-β and *APOEε4* are related to tau pathology, where the interaction between amyloid-β and *APOEε4* is greater than the sum of the independent effects. Voxel-wise analyses revealed a synergistic interaction between *APOEε4* and local [^18^F]AZD4694 SUVR on [^18^F]MK6240 uptake across the cerebral cortex (Fig. 1) independent of age and clinical diagnosis. The interaction between local [^18^F]AZD4694 and *APOEε4* was related to increased [^18^F]MK6240 in the posterior cingulate, precuneus, occipital, and inferior parietal cortices. The results remained similar when employing partial volume corrected data. Scatterplots representing the associations between [^18^F]AZD4694 SUVR and [^18^F]MK6240 SUVR stratified by *APOEε4* genotype are displayed in Supplementary Fig. 1.

**Fig. 2:**
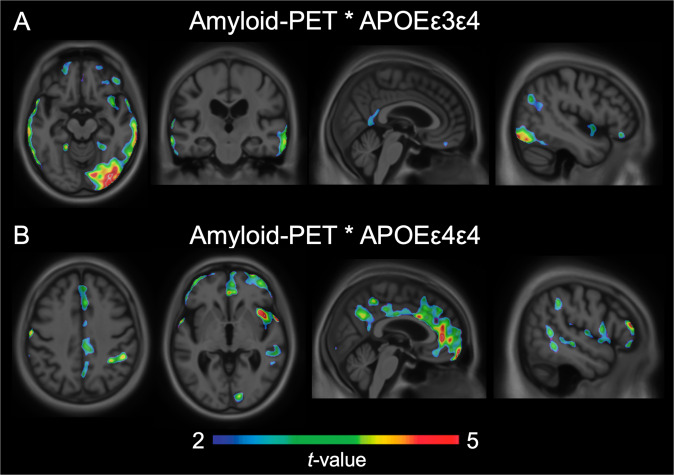
APOEε4 exerts a gene-dose effect on tau aggregation when interacting with amyloid-β. **a** The interaction between [^18^F]Florbetapir and a single *APOEε4* gene was related to increased [^18^F]Flortaucipir uptake in the posterior cingulate posterior parietal, lateral temporal temporooccipital, and orbitofrontal cortices. **b** Homozygous *ε4* carriers demonstrated a more widespread relationship between [^18^F]Florbetapir and [^18^F]Flortaucipir uptake, with [^18^F]Flortaucipir uptake in the posterior cingulate, precuneus, posterior parietal, medial prefrontal, and orbitofrontal cortices. Tau-PET uptake in the temporooccipital cortex was observed only for the interaction between [^18^F]Florbetapir SUVR and one *APOEε4* allele. Effects of homozygosity were observed in the tau-PET uptake in the precuneus, anterior cingulate, and medial prefrontal cortices were observed only for the interaction between [^18^F]Florbetapir SUVR and two *APOEε4* alleles. T-statistical parametric maps were corrected for multiple comparisons using a Random Field Theory cluster threshold of *P* < 0.005, overlaid on the ADNI reference template. Age, clinical diagnosis, and amyloid-β SUVR were employed as covariates in each model. Results remained comparable when using partial volume corrected PET data.

When investigating a gene-dose relationship, different effects were found for *APOEε4* heterozygotes and homozygotes. The interaction between local [^18^F]Florbetapir and one *APOEε4* allele was associated with higher levels of [^18^F]Flortaucipir uptake in posterior cingulate, precuneus, posterior parietal, lateral temporal, temporooccipital, and orbitofrontal cortices. The interaction between local [^18^F]Florbetapir and two *APOEε4* alleles was related to increased [^18^F]Flortaucipir uptake in the posterior cingulate, anterior cingulate, precuneus, posterior parietal, medial prefrontal, and orbitofrontal cortices (Fig. 2). Tau-PET uptake in the temporooccipital cortex was observed only for the interaction between [^18^F]Florbetapir SUVR and one *APOEε4* allele. Effects of homozygosity were observed in the tau-PET uptake in the precuneus, anterior cingulate, and medial prefrontal cortices were observed only for the interaction between [^18^F]Florbetapir SUVR and two *APOEε4* alleles. These effects were independent of age and clinical diagnosis. Again, results remained similar when employing partial volume corrected data. Scatterplots representing the associations between [^18^F]Florbetapir SUVR and [^18^F]Flortaucipir SUVR stratified by *APOEε4* genotype are displayed in Supplementary Fig. 2. When investigating the carrier/noncarrier framework in ADNI (as was conducted in the TRIAD cohort), we observed that the interaction between local [^18^F]Florbetapir and *APOEε4* carriership was associated with higher levels of [^18^F]Flortaucipir uptake in posterior cingulate, precuneus, inferior parietal, lateral temporal temporooccipital, and orbitofrontal cortices (Supplementary Fig. 3).

**Fig. 3:**
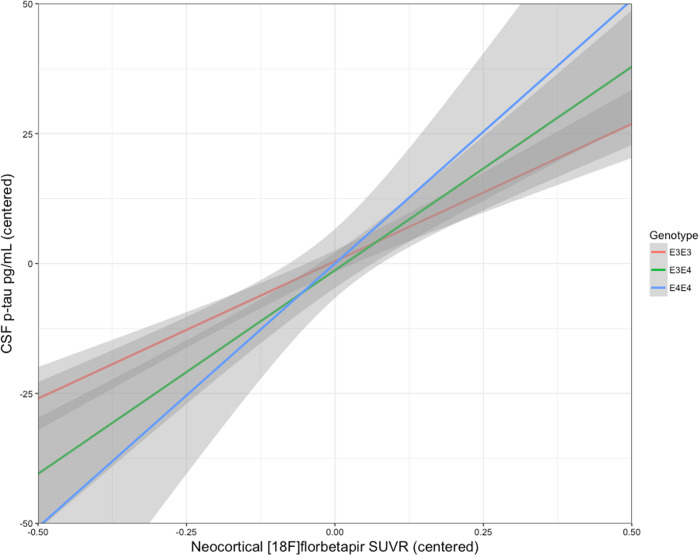
Interaction between amyloid-β and APOEε4 is related to increased CSF phosphorylated tau pathology. The synergistic effect between *APOEε4* and neocortical [^18^F]Florbetapir SUVR was related to increased CSF p-tau. While the heterozygotes (β_4_ = 20.31, se = 6.59, *p* < 0.0001, represented in green) had a milder slope than the homozygotes (β_5_ = 33.01, se = 14.24, *p* = 0.01, represented in blue), this difference in slopes was not statistically significant (*p* = 0.07). Age, clinical diagnosis, and amyloid-β SUVR were employed as covariates.

In a third sample of subjects with CSF measurements of phosphorylated tau, the synergistic effect between *APOEε4* and neocortical [^18^F]Florbetapir SUVR, rather than the sum of their independent effects, was related to increased CSF phosphorylated tau. While the heterozygotes (β_4_ = 20.31, se = 6.59, *p* < 0.0001) had a milder slope than the homozygotes (β_5_ = 33.01, se = 14.24, *p* = 0.01), this difference in slopes was not statistically significant (*p* = 0.07) (Fig. 3). These results were independent of age, clinical diagnosis, and the main effect of amyloid-β.

## Discussion

This study presents in vivo evidence that *APOEε4* potentiates the relationship between amyloid-β and tau pathologies. This potentiation, revealed by the synergistic interaction between *APOEε4* and amyloid-β, was associated with higher levels of tau pathology in the precuneus, posterior cingulate, anterior cingulate, inferior parietal, and basolateral temporal cortices, regions known to exhibit tau accumulation and neurodegeneration across the Alzheimer’s disease spectrum [14, 16, 38, 39]. Homozygous *ε4* carriers had a more widespread pattern of cerebral tau pathology compared with heterozygous *ε4* carriers. The interaction between amyloid-β and one *ε4* allele was related to tau aggregation in the inferior parietal, lateral temporal, orbitofrontal, and posterior cingulate cortices, while the interaction between amyloid-β and two *ε4* alleles was also related to tau aggregation in additional regions including the precuneus, medial prefrontal, and anterior cingulate cortices. In the independent sample of subjects who underwent lumbar puncture, the synergistic interaction between *APOEε4* and amyloid-β on CSF phosphorylated tau was also observed. To the best of our knowledge, this is the first in vivo study demonstrating a synergistic interaction between *APOEε4* and amyloid-β on tau pathology.

**Fig. 4:**
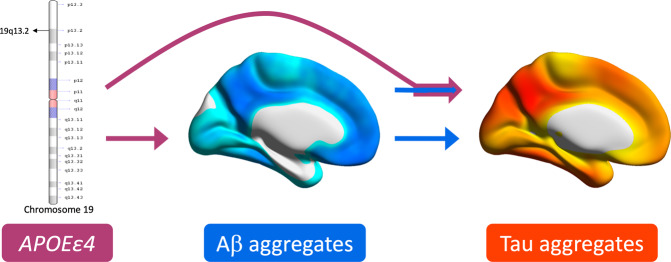
APOEε4 exerts a double hit in Alzheimer’s disease. Schematographic representation of the pathological process presented in this manuscript. APOEε4 exerts a double hit on Alzheimer’s disease risk through its relationship to amyloid-β aggregation, and by potentiating the relationship between amyloid-β and tau pathologies. It is important to note that this figure is intended to illustrate the process described in the present manuscript and is not intended to be a complete description of the roles of APOEε4 or amyloid-β in Alzheimer’s disease.

The effects of *APOEε4* on tau pathology with the presence of amyloid-β may help explain faster disease progression [9, 40] as well as the stronger relationship between amyloid-β and cognitive decline in *APOEε4* carriers [10–12]. Tau-PET uptake in temporal and parietal regions reported in our study are associated with impaired cognitive function [15], and longitudinal studies demonstrated that elevated neocortical tau-PET predicts cognitive decline [41]. Post-mortem studies have also reported that Alzheimer’s disease patients who are *APOEε4* carriers have increased tau pathology compared with noncarriers [42]. Previous studies have also reported a lack of association between primary age-related tauopathy and the *APOEε4* genotype [43], suggesting that the effect of *APOEε4* on tau pathology may be related to its interaction with amyloid-β (Fig. 4). Similarly, post-mortem studies reported that *APOEε4* was associated with increased paired helical filament (PHF) tau in individuals with concomitant amyloid-β pathology, while no association between *APOEε4* and tau was observed in individuals without amyloid-β pathology [44]. Furthermore, recent reports have suggested that amyloid-β synergistically interacts with tau to determine disease progression [45, 46], supporting a framework in which Alzheimer’s disease is characterized by multiple pathological interactions rather than the sequential aggregation of different pathologies.

The brain regions in which tau pathology was related to an amyloid-β × *APOEε4* interaction were concentrated to brain regions known to accumulate tau in Alzheimer’s disease [47]. While both TRIAD and ADNI cohorts demonstrated relationships between amyloid-β × *APOEε4* interactions and tau pathology in the posterior cingulate, precuneus and inferior parietal cortices, small differences between cohorts existed as well. In particular, the medial occipital uptake observed in the TRIAD cohort could be attributable to the AD individuals who also meet criteria for Posterior Cortical Atrophy (PCA), a condition characterized by occipital and posterior parietal tau-PET uptake [15]. Differences in the properties of tau imaging agents could cause the minor differences between cohorts in our manuscript. [^18^F]MK6240 has a 5-fold higher Bmax/Kd (concentration of ﻿available binding sites/equilibrium dissociation constant) ratio than [^18^F]Flortaucipir ﻿in AD brains post-mortem [48]. Correspondingly, it is conceivable that [^18^F]MK6240 captured tau pathology that was below the detection threshold of [^18^F]Flortaucipir. However, head-to-head studies of tau-PET radioligands are needed to clarify this issue as these minor regional discrepancies could also be due to population differences: the TRIAD cohort includes more early onset AD subjects, who have greater cortical tau pathology compared with late onset AD subjects [49].

The present results provide support for an emerging framework in which *APOEε4* exerts pathophysiological effects beyond its involvement in increased cerebral amyloid-β burden [9]. In fact, apoE-immunoreactivity has been demonstrated to aggregate in neurons bearing neurofibrillary tangles [50] and increased expression of *apoE* in neurons is related to increased tau phosphorylation [51–53]. ApoE3, but not apoE4, has been demonstrated to bind to the microtubule-binding repeat region of tau implicated in the self-assembly of tau into PHFs [54], suggesting that there may be isoform-dependent relationships between apoE and tau pathology [55]. Furthermore, truncated apoE4 fragments stemming from stress- or injury-related proteolytic cleavage of apoE are also related to increased tau hyperphosphorylation and neuronal cytoskeletal disruption [56, 57]. *APOEε4* has also been associated with cerebral hypometabolism independently of cerebral amyloid-β burden [58]. Taken together, these studies suggest the need for a reassessment of the role of *APOEε4* throughout the stages of Alzheimer’s disease pathogenesis.

Our study has important methodological limitations. The first is that this study is phenomenological and was not designed to discover a biological mechanism underlying the relationship between *APOEε4*, amyloid-β, and tau. Secondly, despite correcting our analyses for clinical status, longitudinal studies are needed to disentangle whether *APOEε4* carriers had more advanced disease pathophysiology. A methodological strength of the study is the replication of results obtained with a first-generation tau-PET tracer with a second-generation tau-PET tracer [59]. Future work is needed to determine whether the effects of *APOEε4* and amyloid-β on tau result in increased phosphorylation, conformational changes or increased cortical spreading. Because of the different responses of *APOEε4* carriers to disease-modifying pharmaceutical agents [60], a more complete understanding of the involvement of *APOEε4* in Alzheimer’s disease will help guide the development and design of future disease-modifying therapeutic trials.

## Supplementary information

Supplementary table 1

Supplementary Table 2

Supplementary Figure Legends

Supplementary Figure 1

Supplementary Figure 2

Supplementary Figure 3
