# Supplementary material for: APOEε4 potentiates the relationship between amyloid-β and tau pathologies
Source: Mol Psychiatry. 2020 Mar 11;26(10):5977–88. doi: 10.1038/s41380-020-0688-6 (PMC8758492; doi:10.1038/s41380-020-0688-6)
Supplement: Supplementary file 1 — Supplementary table 1 [file 41380_2020_688_MOESM1_ESM.docx]

**Supplementary table 1.** Variance Inflation Factors of variables in statistical models

1. **TRIAD Tau-PET Cohort**

| Brain Region | Variance Inflation Factor (df) |
| --- | --- |
| *APOEε4* | 1.06 (1) |
| Amyloid-PET | 1.91 (1) |
| *APOEε4**Amyloid-PET | 1.58 (1) |
| Age | 1.12 (1) |
| Clinical Diagnosis | 1.51 (2) |

1. **ADNI Tau-PET Cohort**

| Brain Region | Variance Inflation Factor (df) |
| --- | --- |
| *APOEε4* | 1.17 (2) |
| Amyloid-PET | 2.01 (1) |
| *APOEε4**Amyloid-PET | 1.85 (2) |
| Age | 1.11 (1) |
| Clinical Diagnosis | 1.58 (2) |

1. **ADNI CSF Cohort**

| Brain Region | Variance Inflation Factor (df) |
| --- | --- |
| *APOEε4* | 2.28 (2) |
| Amyloid-PET | 1.73 (1) |
| *APOEε4**Amyloid-PET | 3.14 (2) |
| Age | 1.05 (1) |
| Clinical Diagnosis | 1.23 (2) |

This table reports Variance Inflation Factors (VIF) for each variable in each statistical model. Degrees of freedom for each variable are reported in parentheses. Most VIFs were below 2, and all were below 4. A VIF of 1 indicates no collinearity, while VIFs of 5-10 are considered to indicate problematic levels of multicollinearity.
