# Supplementary material for: APOEε4 potentiates the relationship between amyloid-β and tau pathologies
Source: Mol Psychiatry. 2020 Mar 11;26(10):5977–88. doi: 10.1038/s41380-020-0688-6 (PMC8758492; doi:10.1038/s41380-020-0688-6)
Supplement: Supplementary file 2 — Supplementary Table 2 [file 41380_2020_688_MOESM2_ESM.docx]

**Supplementary Table 2.** Standardized main and Interactive effects of Amyloid-PET and *APOEε4* on Tau-PET uptake and CSF p-tau.

1. **TRIAD Tau-PET Cohort**

| Brain Region | Amyloid-PET Main Effect β Estimate (SE) | APOE4 Main β Estimate (SE) | Amyloid-PET * APOE4 Interaction β Estimate (SE) |
| --- | --- | --- | --- |
| Posterior Cingulate | 0.05 (0.06) | -0.05 (0.29) | 0.08 (0.1) |
| Precuneus | 0.03 (0.07) | -0.20 (0.25) | 0.18 (0.11) |
| Inferior Parietal | 0.09 (0.06) | -0.14 (0.25) | 0.13 (0.1) |
| Medial Prefrontal | 0.07 (0.05) | -0.26 (0.21) | 0.22 (0.07) |
| Occipital | 0.05 (0.05) | -0.44 (0.19) | 0.22 (0.07) |

1. **ADNI Tau-PET Cohort**

| Brain Region | Amyloid-PET Main β Estimate (SE) | Single APOE4 Main β Estimate (SE) | Amyloid-PET * Single APOE4 Interaction β Estimate (SE) |
| --- | --- | --- | --- |
| Posterior Cingulate | 0.05 (0.02) | -0.04 (0.03) | 0.09 (0.03) |
| Lateral Temporal | 0.12 (0.02) | -0.04 (0.03) | 0.11 (0.03) |
| Inferior Parietal | 0.08 (0.02) | -0.02 (0.04) | 0.11 (0.04) |
| Orbitofrontal | 0.07 (0.02) | -0.05 (0.04) | 0.08 (0.03) |
| Temporooccipital | 0.08 (0.02) | -0.01 (0.03) | 0.15 (0.04) |

1. **ADNI Tau-PET Cohort**

| Brain Region | Amyloid-PET Main β Estimate (SE) | Double APOE4 Main β Estimate (SE) | Amyloid-PET * Double APOE4 Interaction β Estimate (SE) |
| --- | --- | --- | --- |
| Posterior Cingulate | 0.09 (0.02) | -0.07 (0.06) | 0.15 (0.05) |
| Lateral Temporal | 0.07 (0.02) | -0.002 (0.06) | 0.16 (0.05) |
| Inferior Parietal | 0.08 (0.02) | 0.004 (0.07) | 0.2 (0.06) |
| Medial Prefrontal | 0.06 (0.02) | -0.07 (0.05) | 0.12 (0.04) |
| Occipital  Orbitofrontal  Dorsolateral Prefrontal | 0.07 (0.02)  0.09 (0.02)  0.07 (0.02) | -0.01 (0.05)  -0.07 (0.05)  -0.01 (0.06) | 0.13 (0.04)  0.17 (0.05)  0.18 (0.05) |

1. **ADNI Lumbar Puncture Cohort**

|  | Amyloid-PET Main Effect β Estimate (SE) | Single APOE4 Main Effect β Estimate (SE) | Amyloid-PET * Single APOE4 Interaction Effect β Estimate (SE) |
| --- | --- | --- | --- |
| CSF p-tau | 3.06 (0.73) | 4.2 (1.36) | 4.2 (1.36) |

1. **ADNI Lumbar Puncture Cohort**

|  | Amyloid-PET Main Effect β Estimate (SE) | Double APOE4 Main Effect β Estimate (SE) | Amyloid-PET * Double APOE4 Interaction Effect β Estimate (SE) |
| --- | --- | --- | --- |
| CSF p-tau | 3.06 (0.73) | 3.89 (1.28) | 6.83 (2.94) |

Supplementary table 2 reports standardized beta coefficients for main and interactive effects of Amyloid-PET and *APOEε4* on tau. 2 A-C: standardized beta coefficients from brain regions where a significant synergistic effect of Amyloid-PET and *APOEε4* on Tau-PET was observed. D,E: Standardized beta coefficients from global neocortical Amyloid-PET and *APOEε4* on CSF p-tau. Standard errors are reported in parentheses.
