# Supplementary material for: APOEε4 potentiates the relationship between amyloid-β and tau pathologies
Source: Mol Psychiatry. 2020 Mar 11;26(10):5977–88. doi: 10.1038/s41380-020-0688-6 (PMC8758492; doi:10.1038/s41380-020-0688-6)
Supplement: Supplementary file 3 — Supplementary Figure Legends [file 41380_2020_688_MOESM3_ESM.docx]

**Supplementary Figure 1: Associations between Amyloid-PET and Tau-PET stratified by APOEε4 status in TRIAD.**

Scatterplots showing associations between regional Amyloid-PET SUVR and Tau-PET SUVR from the TRIAD cohort. SUVRs are extracted from RFT-corrected significant clusters derived from voxel-wise analyses (see Figure 1 and Table 2).

**Supplementary Figure 2: Associations between Amyloid-PET and Tau-PET stratified by APOEε4 status in ADNI.**

Scatterplots showing associations between regional Amyloid-PET SUVR and Tau-PET SUVR from the ADNI cohort. SUVRs are extracted from RFT-corrected significant clusters derived from voxel-wise analyses (see Figure 2 and Table 2).

**Supplementary Figure 3: APOEε4 carrier / noncarrier framework in ADNI subjects.**

When investigating the *APOEε4* carrier/noncarrier framework in ADNI subjects (in contrast to the dose-dependent framework), we observed significant associations between the *APOEε4**Amyloid-PET interaction and [^18^F]Flortaucipir SUVR in the posterior cingulate, precuneus, lateral temporal, inferior parietal and medial prefrontal cortices. T-statistical parametric maps were corrected for multiple comparisons using Random Field Theory cluster threshold of *P* < 0.005, overlaid on the ADNI reference template. Age, clinical diagnosis and amyloid-β SUVR were employed as covariates in each model. Results remained comparable when using partial volume corrected PET data.

**Supplementary table 1.** Variance Inflation Factors of variables in statistical models

This table reports Variance Inflation Factors (VIF) for each variable in each statistical model. Degrees of freedom for each variable are reported in parentheses. Most VIFs were below 2, and all were below 4. A VIF of 1 indicates no collinearity, while VIFs of 5-10 are considered to indicate problematic levels of multicollinearity.

**Supplementary Table 2.** Standardized main and Interactive effects of Amyloid-PET and *APOEε4* on Tau-PET uptake and CSF p-tau.

Supplementary table 2 reports standardized beta coefficients for main and interactive effects of Amyloid-PET and *APOEε4* on tau. A-C: standardized beta coefficients from brain regions where a significant synergistic effect of Amyloid-PET and *APOEε4* on Tau-PET was observed. D,E: Standardized beta coefficients from global neocortical Amyloid-PET and *APOEε4* on CSF p-tau. Standard errors are reported in parentheses.
