# Supplementary figures and images for: APOEε4 potentiates the relationship between amyloid-β and tau pathologies
Source: Mol Psychiatry. 2020 Mar 11;26(10):5977–88. doi: 10.1038/s41380-020-0688-6 (PMC8758492; doi:10.1038/s41380-020-0688-6)

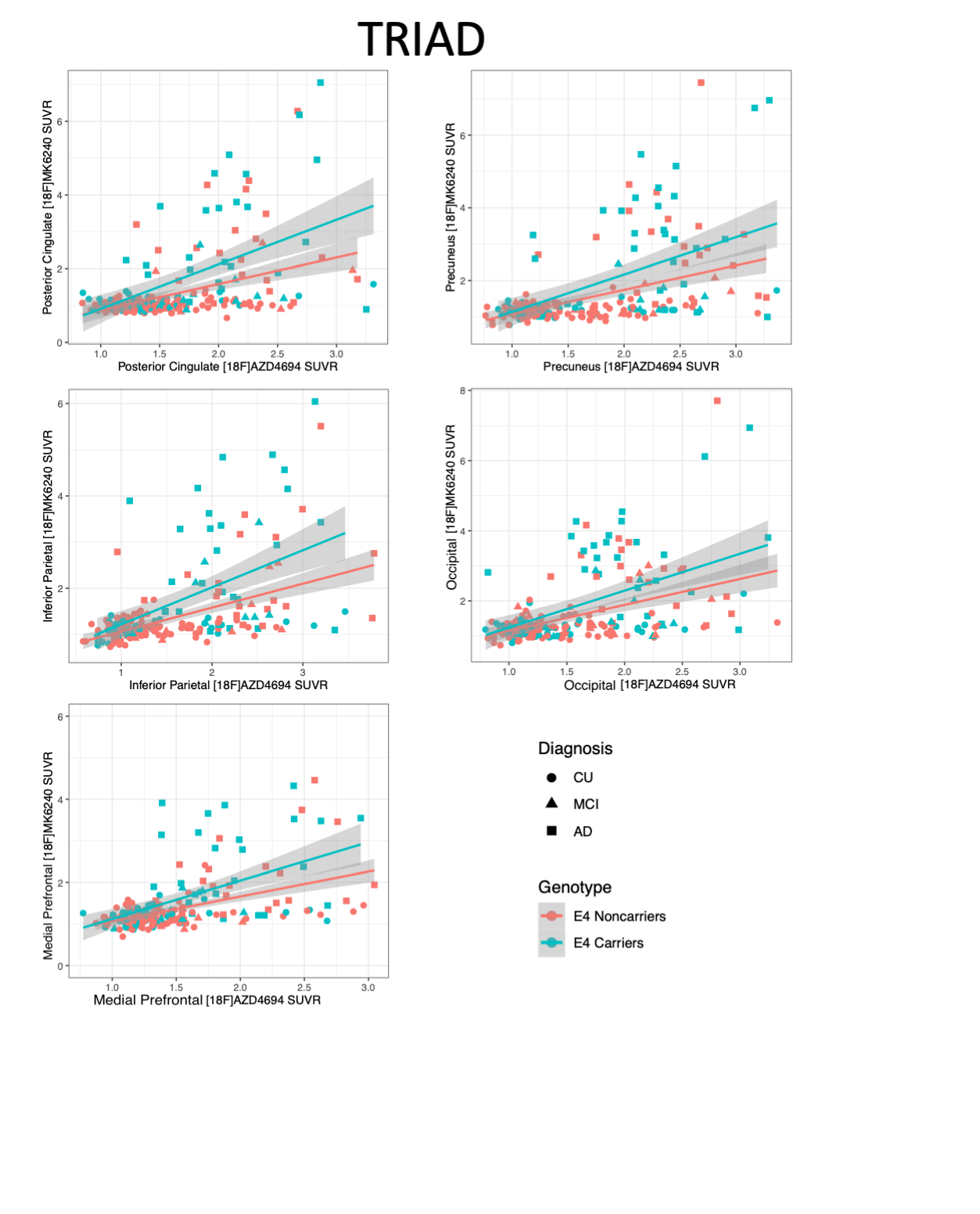

Supplement: Supplementary file 4 — Supplementary Figure 1 [file 41380_2020_688_MOESM4_ESM.tif]

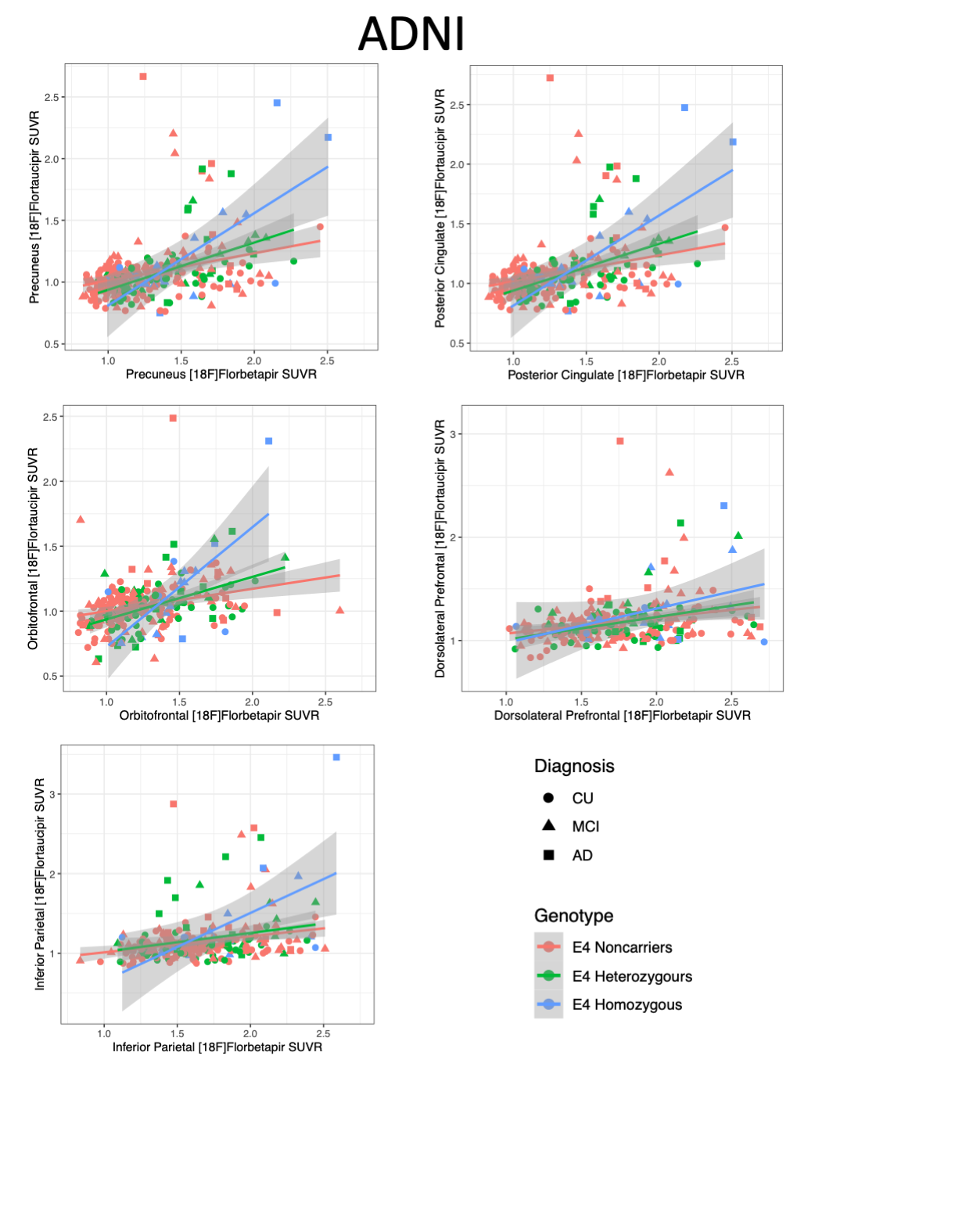

Supplement: Supplementary file 5 — Supplementary Figure 2 [file 41380_2020_688_MOESM5_ESM.tif]

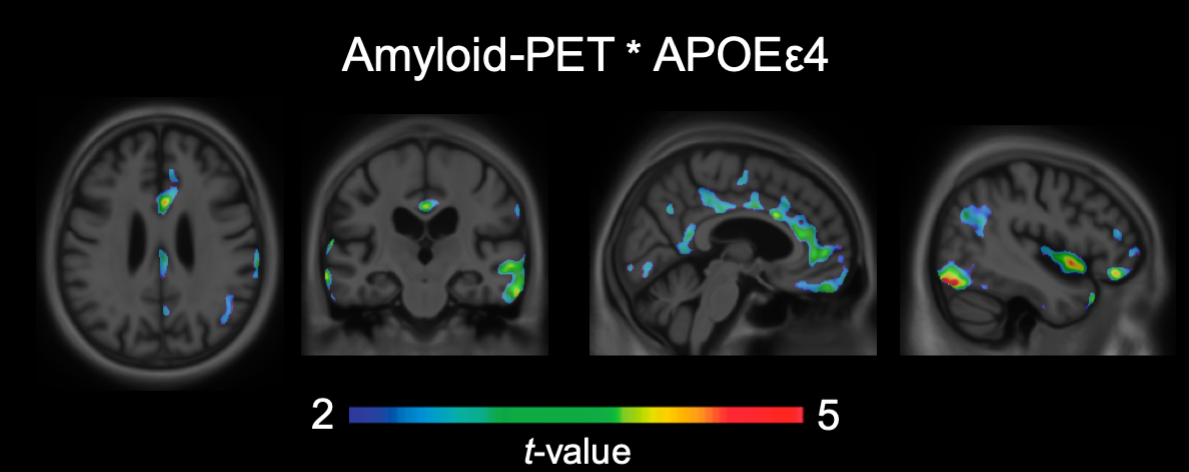

Supplement: Supplementary file 6 — Supplementary Figure 3 [file 41380_2020_688_MOESM6_ESM.tif]
